# The prevalence of CGG repeat expansion mutation in *FMR1* gene in the northern Chinese women of reproductive age

**DOI:** 10.1186/s12881-019-0805-z

**Published:** 2019-05-16

**Authors:** Yinan Ma, Xing Wei, Hong Pan, Songtao Wang, Xin Wang, Xiaowei Liu, Liying Zou, Xiaomei Wang, Xiaorong Wang, Hua Yang, Fengying Wang, Kefang Wang, Lifang Sun, Xiaolin Qiao, Yue Yang, Xiuhua Ma, Dandan Liu, Guifeng Ding, Junqi Ma, Xiuli Yang, Sainan Zhu, Yu Qi, Chenghong Yin

**Affiliations:** 1 Department of Central Laboratory, Peking University First Hospital, Beijing, 100034 China; 2 Beijing Huanuo Aomei Gene Biotech Co. Ltd., Beijing, 100070 China; 3 Department of Obstetrics & Gynecology, Beijing Obstetrics and Gynecology Hospital, Beijing, 100006 China; 4 Department of Obstetrics & Gynecology, Beijing Friendship Hospital, Capital Medical University, Beijing, 100050 China; 5 Department of Obstetrics & Gynecology, Xuanwu Hospital, Capital Medical University, Beijing, 100053 China; 6 Department of Obstetrics & Gynecology, Beijing Anzhen Hospital, Capital Medical University, Beijing, 100029 China; 7 Department of Obstetrics & Gynecology, Beijing Jishuitan Hospital, Beijing, 100035 China; 8 Department of Obstetrics & Gynecology, Beijing Chaoyang District Maternal and Child Health Care Hospital, Beijing, 100022 China; 9 Department of Obstetrics & Gynecology, Civil Aviation General Hospital, Beijing, 100025 China; 10 Department of Obstetrics & Gynecology, People’s Hospital of Beijing Daxing District, Beijing, 102600 China; 11 Department of Obstetrics & Gynecology, Beijing Changping Hospital, Beijing, 102200 China; 12 Department of Obstetrics & Gynecology, Xinjiang Urumqi City Maternal and Child Care Health Hospital, Urumqi, 830001 China; 13 Department of Obstetrics &Gynecology, The First Affiliated Hospital of Xinjiang Medical University, Urumqi, 830054 China; 14 Department of Obstetrics &Gynecology, Peking University First Hospital, Beijing, 100034 China; 15 Department of Statistics, Peking University First Hospital, Beijing, 100034 China; 16 Department of Prenatal Diagnosis Center, Beijing Obstetrics and Gynecology Hospital, Capital Medical University, Beijing, 100026 China

**Keywords:** China, Fragile X syndrome, *FMR1* gene, Pre-mutation, abortion

## Abstract

**Background:**

The prevalence of CGG repeat expansion mutation in *FMR1* gene varies among different populations. In this study, we investigated the prevalence of this mutation in women of reproductive age from northern China.

**Methods:**

A total of 11,891 pre-conceptional or pregnant women, including 5037 pregnant women and 7357 women with the history of spontaneous abortion or induced abortion due to delayed growth of the embryos, were recruited. The number of CGG repeats in *FMR1* was measured by the TRP-PCR method. We also offered genetic counseling and prenatal diagnosis to the women carrying pre-mutation or full mutation alleles.

**Results:**

The prevalence of pre-mutation in reproductive women in northern China was 1/410, higher than that in southern China and Korea but lower than that in western countries. We also found that the prevalence of pre-mutation was relatively high (1/320) in women with abortion history.

**Conclusion:**

Screening for CGG repeat expansion mutation in *FMR1* should be recommended to the women with the history of spontaneous abortion or induced abortion due to delayed growth of the embryos.

## Background

Fragile X syndrome (FXS, OMIM 300624), one of the common forms of familial intellectual disability, is caused by CGG repeat expansion in the 5′-untranslated region of *FMR1* gene on X chromosome. According to the standards and guidelines for fragile X testing from American College of Medical Genetics (ACMG), 5–44 CGG repeats can be defined as normal, 45–54 CGG repeats as intermediate or in a grey zone, 55–200 CGG repeats as pre-mutation, and > 200 CGG repeats as full mutation [1, 2].

Individuals with the full mutation are typically with FXS. Pre-mutation carriers are not associated with FXS, but have an increased risk for fragile X associated primary ovarian insufficiency (FXPOI) or fragile X associated tremor/ataxia syndrome (FXTAS). Female pre-mutation carriers have a higher chance to have FXS children because of the potential repeat instability of pre-mutation allele after transmission. Intermediate carriers are at a higher risk for expanding of CGG repeats to give pre-mutation offspring or full mutation patients in subsequent generations [3, 4]. Therefore, identification of pre-mutation in women of reproductive age is of clinically significance for providing information about the risk for FXPOI and the birth of FXS children [4–6].

The screening for CGG expansion mutation in *FMR1* has been conducted in many countries [7–11]. The prevalence of CGG repeat pre-mutation varies in different populations but is unknown in northern China. In this study, we demonstrate the prevalence of CGG repeat mutation in *FMR1* in a cohort of 11,891 women of reproductive age from northern China as well as pregnancy outcome in the pre-mutation and full mutation carriers in this cohort.

## Methods

### Subjects

A total of 11,891 pre-conceptional or pregnant women from obstetrics department or family planning department were tested at the Central Laboratory of Peking University First Hospital during the period from Jan. 2015 to Sep. 2017. They asked for the test after the education and genetic counseling from doctors. A part of them had the history of spontaneous abortion or induced abortion due to delayed growth of the embryos. The family history of mental retardation was excluded by questionnaire. Informed consent was obtained from these women. This study was approved by the Medical Ethics Committee of Peking University First Hospital.

Their age ranged from 21 to 50 years (31.33 ± 5.87 years). Among these women, 5037 were pregnant (12–22 weeks), 7357 had the history of spontaneous abortion or induced abortion due to delayed growth of the embryos, and 4534 had no specific history. After the test, the women with pre-mutation or full mutation alleles were advised to take prenatal diagnosis for CGG repeats in *FMR1* in amniotic fluid, chorionic villi or cord blood when they became pregnant.

### Laboratory methods

The number of CGG repeats in *FMR1* was measured by triplet repeat primed PCR (TRP-PCR) using the Amplide X *FMR1* PCR Kit (Asuragen, Austin, TX, USA) following the manufacturer protocol. This method can detect the CGG repeats from 8 to > 200. Total DNA was extracted from peripheral leukocytes by routine method. PCR was performed in an ABI GeneAmp PCR system 9700 thermal cycler (Applied Biosystems, Foster City, CA, USA). Amplicon was sized on an ABI 3730xl Genetic Analyzer (Applied Biosystems) and analyzed using GeneMapper 4.0 software (Applied Biosystems). Number of CGG repeats in *FMR1* was then categorized as normal (< 45 repeats), intermediate (45–54 repeats), pre-mutation (55–200 repeats) or full mutation (> 200 repeats).

For prenatal diagnoses, DNA were extracted from chorionic villi, amniotic fluid or cord blood using the DNeasy Blood & Tissue Kit (Qiagen, Hilden, Germany) and the same TRP-PCR method. Linkage analyses were also included using five STR markers nearby *FMR1* to exclude false results due to maternal blood contamination.

The prevalence of CGG repeat mutation was defined as the ratio of pre-mutation and full mutation alleles to total *FMR1* alleles. The 95% confidence interval (CI) was calculated using Wilson score interval method. Chi-square test was used to compare the groups with and without abortion history. A *P* value < 0.05 was considered to be statistically significant.

## Results

A total of 11,819 women of reproductive age from northern China were screened for CGG repeat expansion mutation in *FMR1*. The number of this CGG repeats was normally 29, encompassing 42.5% alleles of this cohort. Intermediate mutation carriers (45–54 CGG repeats), pre-mutation carriers (55–200 CGG repeats) and full mutation carriers (> 200 CGG repeats) were found in 76, 29 and 3 women, respectively, with the prevalence of 1/156 (CI 1:199~125), 1/410 (CI 1:588~286) and 1/3940 (CI 1:11,765~1351), respectively. Table 1 shows the distribution of CGG repeat number in the 32 pre-mutation and full mutation carriers.

**Table 1:** Distribution of CGG repeat number in the 29 pre-mutation carriers and 3 full mutation carriers

Abortion history	Number of CGG repeats							
	55–60	61–65	66–70	71–75	76–80	81–200	> 200	Total
Negative	1	0	1	0	3	1	2	8
Positive	5	4	3	0	1	10	1	24
Total	6	4	4	0	4	11	3	32

The relationship between CGG repeat mutation carrier and abortion history is present in Table 2, which indicates that the prevalence of pre-mutation carriers was higher in women with abortion history (1/320) than in those without abortion history (1/756), but without statistically significance (*P* = 0.053).

**Table 2:** Relationship between CGG repeat mutation carrier and abortion history

CGG repeat mutation carrier	Abortion history		*P* value
	Negative (*n* = 4534)	Positive (*n* = 7357)	
Intermediate	32(0.7%)	44(0.6%)	X^2^ = 0.512, *P* = 0.474
Pre-mutation	6(0.13%)	23(0.31%)	X^2^ = 3.748, *P* = 0.053
Full mutation	2(0.04%)	1(0.01%)	*P* = 0.561*

^*^Fisher’s exact test

In the 17 pregnant mothers of pre-mutation or full mutation carriers, 15 mothers agreed to perform prenatal diagnosis of the fetuses. Table 3 shows the prenatal diagnosis results. In the 13 pre-mutation mothers, 8 fetuses carried expanded CGG repeats from their mothers (CGG repeats = 78~115) and became full mutation carriers; 2 fetuses inherited the pre-mutation allele from their mothers with only minor expansion of the CGG repeats; and 3 fetuses inherited the normal *FMR1* alleles from their mothers. In the 2 full mutation mothers, one fetus inherited the full-mutation allele (CGG > 200) from mother and was also a 21-trisomy (47, XY + 21) by karyotyping; the other fetus fortunately inherited the normal allele (CGG repeats = 36) from mother.

**Table 3:** Results of prenatal diagnosis in pregnant mothers of pre-mutation and full mutation carriers

Case no.	Number of CGG repeats Mother	Fetus	Outcome of pregnancy
1	78/28	> 200	Terminated
2	78/36	36	Continue
3	91/29	> 200	Terminated
4	85/31	94	Continue
5	56/29	29	Continue
6	95/33	33	Continue
7	91/29	> 200	Terminated
8	98/29	> 200	Terminated
9	115/36	> 200	Terminated
10	89/29	> 200	Terminated
11	79/29	> 200	Terminated
12	69/29	79/32	Continue
13	81/30	> 200	Terminated
14	> 200/36	36/29	Continue
15	> 200/32	> 200 & 21-trisomy	Terminated

We also found a 32 years old woman carrying mosaic full mutation (CGG repeats = 200), pre-mutation (CGG repeats = 97) and normal CGG repeats (CGG repeats = 30). She had no neurological symptoms, menstrual irregularities or sex hormone problems but had the history of one abortion.

## Discussion

To the best of our knowledge, this is the first study on the prevalence of CGG repeat expansion mutation in *FMR1* in women of reproductive age in China. In this cohort, the prevalence of CGG repeat expansion mutation in *FMR1* was 1/410 for pre-mutation and 1/3964 for full mutation. Totally, the prevalence of pre-mutation and full-mutation was 1/372, which is useful to estimate the risk for FXS transmitted from mothers.

Many researches have shown that the founder effect is partially responsible for the variation of carrier frequency in different populations. The prevalence of pre-mutation carrier in women of reproductive age without family history of intellectual disability or with unselected family history was 1/151–382 in USA [12–15], 1/259–549 in Canada [16], 1/83 in Finland [17] and 1/113–157 in Israel [18, 19]. However, the prevalence was lower in eastern countries. In a study of 5470 Korean women of reproductive age without family history, the prevalence of pre-mutation carrier was 1/781 [20, 21]. Two recent studies on the pregnant women from Hong Kong and Taiwan in southern China showed the prevalence of 1/1325 (total subjects = 2650) and 1/1955 (total subjects = 3911), respectively [22–24]. The prevalence of our cohort (1/410) is higher than that from Korea, Hong Kong and Taiwan, and is similar to that from Korea if we exclude the women with the history of spontaneous abortion or induced abortion due to delayed growth of the embryos from total subjects (1/756).

In this cohort, the prevalence of pre-mutation carriers was higher in women with the history of spontaneous abortion or induced abortion due to delayed growth of the embryos than in those without abortion history (1/320 vs. 1/756, *P* = 0.053), of which the information was not found in the literature. Totally, 61.2% of our subjects had abortion history. The prevalence of pre-mutation carrier of our subjects was 1/410, higher than that in other eastern countries. More samples should be tested to confirm the difference because of only 29 pre-mutation carriers we detected in this cohort.

The mechanism of abortion associated with pre-mutation of CGG repeat is not clear. In addition to FXPOI, pre-mutation carrier women are also at higher risks for the abnormalities involving neurology, reproductive, endocrinology, immunology and psychiatry systems [25]. Pre-mutation carrier women present a continuum of diminished ovarian follicular reserve, from which irregularity of menstrual cycle, decreased fertility, fluctuation of hormone levels, poor quality of oocytes in follicles and abortion occur [25–27].

Due to the low incidence of CGG repeat expansion mutation in *FMR1*, it is not recommended to widely screen for this mutation in population in China. The guidelines for fragile-X test proposed by American Congress of Obstetricians and Gynecologists (ACOG) and American College of Medical Genetics (ACMG) [1, 3] recommend that this test should be performed in individuals with a personal or family history of fragile-X, fragile-X-related disorders, unexplained mental retardation or developmental delay, autism, ovarian insufficiency or elevated follicle-stimulating hormone before 40 years old of unknown cause. Because the prevalence of pre-mutation carriers is relatively high in women with abortion history in this cohort, the indications for screening CGG repeat mutation should include women having the history of spontaneous abortion or induced abortion due to delayed growth of the embryos.

In this study, 15 of the 17 pregnant women accepted prenatal diagnosis for the fetuses. All of the 9 mothers having full mutation fetuses agreed to terminate the pregnancy. In the 13 pregnant women with pre-mutation, the pre-mutation alleles were transmitted to 10 fetuses, in which 8 pre-mutation alleles with the CGG repeats of 78–115 expanded greatly to full mutation alleles. Our results are consistent with the previous notion that 55 (or 60) CGG repeats can be used as the cutoff value. Prenatal diagnosis is essential if the pregnant woman brings CGG repeats in *FMR1* more than the cutoff value. Two of the 17 pregnant women with pre-mutation (60 and 69 CGG repeats, respectively) refused to perform prenatal diagnosis and had normal babies by follow-up study. But there was a limitation for this study that the methylation of status for the full mutation was not detected.

Some women with the indications were not willing to do CGG repeat expansion test due to medical expense problems. In addition, women with abortion were more likely to try this test. The sample bias impacts on the prevalence of pre-mutation carrier in this cohort.

## Conclusions

The prevalence of pre-mutation in reproductive women from northern China was 1/410, higher than that in southern China and Korea but lower than that in western countries. The prevalence of pre-mutation was higher (1/320) in the reproductive women with the history of spontaneous abortion or induced abortion due to delayed growth of the embryos. Therefore, screening for CGG repeat expansion mutation in *FMR1* should be recommended to the women with the history of spontaneous abortion or induced abortion due to delayed growth of the embryos.

## Abbreviations

ACMG: American College of Medical Genetics
*FMR1*: Fragile X mental retardation 1 gene
FXPOI: Fragile X associated primary ovarian insufficiency
FXS: Fragile X syndrome
FXTAS: Fragile X associated tremor/ataxia syndrome
TRP-PCR: Triplet repeat primed PCR
